# Analysis of Medaka *sox9* Orthologue Reveals a Conserved Role in Germ Cell Maintenance

**DOI:** 10.1371/journal.pone.0029982

**Published:** 2012-01-12

**Authors:** Shuhei Nakamura, Ikuko Watakabe, Toshiya Nishimura, Atsushi Toyoda, Yoshihito Taniguchi, Minoru Tanaka

**Affiliations:** 1 Laboratory of Molecular Genetics for Reproduction, National Institute for Basic Biology, Okazaki, Japan; 2 Department of Basic Biology, The Graduate University for Advanced Studies (SOKENDAI), Okazaki, Japan; 3 Comparative Genomics Laboratory, National Institute of Genetics, Mishima, Japan; 4 Department of Preventive Medicine and Public Health, School of Medicine, Keio University Shinanomachi 35, Tokyo, Japan; National University of Singapore, Singapore

## Abstract

The sex determining gene is divergent among different animal species. However, *sox9* is up-regulated in the male gonads in a number of species in which it is the essential regulator of testis determination. It is therefore often discussed that the sex determining gene-*sox9* axis functions in several vertebrates. In our current study, we show that *sox9b* in the medaka (*Oryzias latipes*) is one of the orthologues of mammalian *Sox9* at syntenic and expression levels. Medaka *sox9b* affects the organization of extracellular matrices, which represents a conserved role of *sox9*, but does not directly regulate testis determination. We made this determination via gene expression and phenotype analyses of medaka with different copy numbers of *sox9b*. *Sox9b* is involved in promoting cellular associations and is indispensible for the proper proliferation and survival of germ cells in both female and male medaka gonads. Medaka mutants that lack *sox9b* function exhibit a seemingly paradoxical phenotype of sex reversal to male. This is explained by a reduction in the germ cell number associated with aberrant extracellular matrices. Together with its identified roles in other vertebrate gonads, a testis-determining role for *Sox9* in mammals is likely to have been neofunctionalized and appended to its conserved role in germ cell maintenance.

## Introduction

*Sox9* is a member of the *Sry*-related HMG box (*Sox*) gene family and is conserved in vertebrates. Among the mammalian *Sox* family, *Sox9* has been extensively analyzed and is known to be critical for many aspects of cell differentiation such as chondrocyte specification, neural crest differentiation, heart valve development and male sex determination [1]–[4]. Many of these functions are achieved through the role of *sox9* in the extracellular matrix and this has been confirmed in various vertebrates. This suggests that *sox9* is conserved both structurally and functionally.

During the initial events in sex determination, many vertebrates employ a species-specific sex determining gene. In mammals, the sex determining gene, *Sry*, is on the Y chromosome and directly upregulates the transcription of *Sox9* in the supporting cells of the XY gonad only. Once *Sox9* expression is established in the XY supporting cells, it is both functionally required and sufficient for testis determination [5]–[9]. In other higher animals, such as the chicken, alligator and turtle, *sox9* is also up-regulated exclusively in the male gonad [10]–[12]. In lower vertebrates, the role of sox9 in the gonad is not yet known. It has been often described that a sex determining gene-*sox9* axis may constitute a conserved component of the sex determination system in many vertebrates.

The medaka (*Oryzias latipes*) is a species of teleost fish and represents a good model system for studying the conserved mechanisms of sex determination and the differentiation of gonads across different vertebrate species [13]. Male sex determination in the medaka is initiated in the supporting cells via the expression of the DM-domain gene on the Y chromosome, *DMY*/*dmrt1bY* [14], [15]. However, the involvement of the medaka *sox9* homologue in *DMY*/*dmrt1bY*-involving testis determination has not yet been functionally addressed.

As a result of teleost-specific genome duplication [16], most genes in teleosts are present in two copies. Two *sox9* genes have also been reported in the medaka genome, *sox9a* and *sox9b*/*sox9a2* (hereafter referred to as *sox9b*) [17]–[19]. Among these two copies, *sox9a* is not expressed in the gonadal supporting cells essential for sex determination, but is expressed in oocytes of the adult ovary [19]. In contrast, *sox9b* initiates its expression in the gonadal precursor cells that develop into the supporting cells [20]. It is therefore thought that the expression of *sox9b* in the supporting cells of the medaka parallels the role of mammalian *Sox9* in the gonads.

Our previous examinations have revealed that unlike mammalian gonads, *sox9b* expression in the medaka is maintained in a few supporting cells in the developing ovary. In addition, the histological units of *sox9b*-expressing supporting cells in medaka have been recently identified as ovarian niche structures (known as the germinal cradles) that contain germline stem cells [21]. These expression patterns led us to speculate that the role of medaka *sox9b* may differ from that of mammalian *Sox9* during testis determination.

Using syntenic and gene expression analysis in our current study, we first reconfirmed that medaka *sox9a* and *sox9b* are co-orthologues of mammalian *Sox9* and that *sox9b*, but not *sox9a*, is expressed in the supporting cells responsible for initiation of medaka testis determination [17], [22]. Using both transgenic and chimeric *sox9b* medaka mutants, we show that medaka *sox9b* is required for germ cell proliferation and survival, but not for testis determination. The expression of components of the extracellular matrix was found to be largely disorganized in *sox9b* mutant medaka gonads. In addition, our results show that zebrafish *sox9a* is also expressed in the ovarian supporting cells. These findings collectively challenge the discussion along with the mammalian *Sox9* function in the gonads and suggest that a testis determining role is an appended function during vertebrate evolution.

## Results

### Syntenic analysis and expression study of medaka *sox9* genes

To confirm the phylogenetic relationships between medaka *sox9a* and *sox9b* with other vertebrate *sox9* genes, we first examined the *sox9* synteny among representative vertebrates using the *Ensemble* genomic information (Fig. S1A). As expected from previous analysis [17], the genomic region around *sox9* is conserved in the mouse, chick and frog (*Xenous tropicalis*) and is duplicated in the teleosts stickleback, medaka and zebrafish. The two regions in the teleosts contain either *sox9a* or *sox9b*. Other than these two genes, no other *sox9*-like gene could be found using whole genome scanning. In addition to these observations, the main *Sox9* expression domains in mammalian embryos have been shown to correspond to both or either *sox9a* and/or *sox9b* in teleost embryos. Hence, *sox9a* and *sox9b* in teleosts are very likely to be the only co-orthologues of the *sox9* gene in other vertebrates [17], [22].

Our current expression analysis demonstrated that medaka *sox9a* is not expressed in the somatic cells during gonadal differentiation (*sox9a*, XY n = 5, XX n = 5). *Sox9b* was only detected in the supporting cells surrounding the germ cells (*sox9b*, XY n = 5, XX n = 5) (Fig. S1B), as expected from previous reports [18]–[20]. These results may be supportive of the conventional view that medaka *sox9b* has a conserved role in sex determination in the supporting cells within the medaka sex determining gene pathway. However, unlike mammalian *Sox9* which is expressed in male supporting cells only, medaka *sox9b* is also expressed in the female developing gonads [20]. Additionally, in the adult ovary, *sox9b*-expressing supporting cells constitute niche structures harboring germline stem cells [21].

To evaluate whether the female expression of *sox9* is specific to the medaka, we examined the gonadal *sox9a* and *sox9b* expression patterns of a phylogenetically distant teleost, the zebrafish. The previous reports indicate that zebrafish *sox9a* is expressed in the male supporting cells in adult testes (Fig. S2C) [22], [23]. Our present *in situ* hybridization analyses revealed that zebrafish *sox9a* expression occurs in some populations of somatic cells surrounding the small germ cells in the adult ovaries (Fig. S2A, S2B) while zebrafish *sox9b* was found to be expressed in the oocytes of adult ovaries but not in the testes (Fig. S2D, S2E). The expression analysis, suggests that there is functionally conserved similarity between medaka *sox9b* and zebrafish *sox9a* in gonads. This, together with our syntenic analysis, supports the previous report by Klüver et al that medaka *sox9a* and *sox9b* are an example of lineage-specific subfunctionalization and have arisen from duplication of the ancestral proto-chromosome 2 [17]. We next conducted functional analysis of the medaka *sox9b* gene to reveal a possible conserved role in teleost gonads.

### Identification of *sox9b* mutants

**Figure 1:**
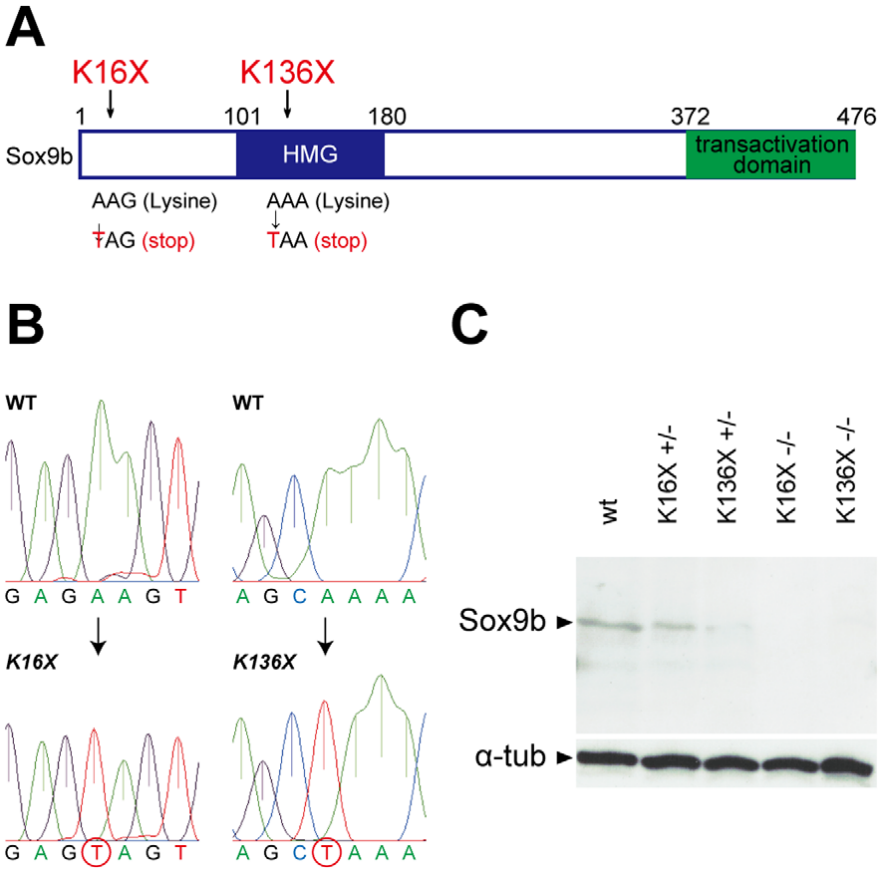
Identification and characterization of two *sox9b* mutant alleles in medaka. (A) Schematic representation of the sox9b medaka protein and the locations of mutations. (B) Genomic sequences of wild-type *sox9b*, and of the *sox9b^K16X^* (−/−) and *sox9b^K136X^* (−/−) mutants. (C) Western blotting analysis of sox9b and alpha-tubulin demonstrating the absence of expression in the homozygous mutants, confirming that both alleles are null.

To address the function of *sox9b* in medaka gonads, we isolated two nonsense mutant alleles, *sox9b^K16X^* and *sox9b^K136X^* from the medaka tilling library (Fig. 1A, 1B) [24]. These variants have nonsense mutations in the 5′ coding region and HMG box domain, respectively. Western blotting analysis further revealed that the sox9b protein product levels are absent in the homozygous mutants (Fig. 1C), indicating that both of the nonsense alleles are functionally null. The heterozygous mutants of both alleles were found to be viable and to reach sexual maturity, although their reproductive ability was low. However, both the *sox9b^K16X^* and *sox9b^K136X^* homozygous mutants died by 20 dph (days post hatching) which is approximately 28 dpf (days post fertilization).

### *Sox9b* is not required for testis determination

**Figure 2:**
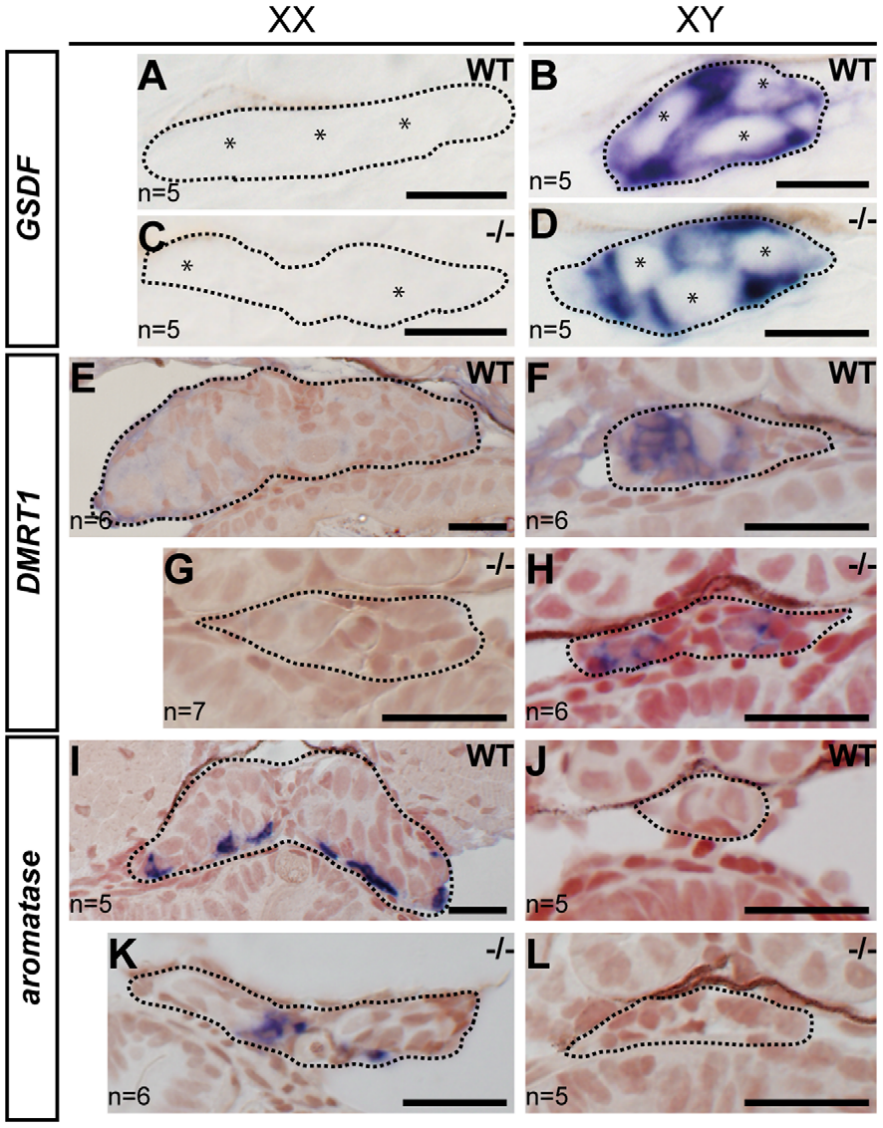
Early stage sex differentiation processes are unaffected in the *sox9b* mutant gonads. (A–L) *GSDF* (A–D), *DMRT1* (E–H) and *aromatase* (I–L) expression (blue) were detected by in situ hybridization analysis of st. 39 (A–D) and 10 dph (E–L) medaka embryos, respectively. Consistent with the pattern found in wild-type (WT) medaka gonads, *GSDF* and *DMRT1* were found to be expressed in XY gonads of the homozygous *sox9b* mutants, whilst *aromatase* was expressed in XX gonads of the homozygous *sox9b* mutants. More than five gonads were examined in each experiment. Dotted lines (A–L), gonadal outlines; asterisks in (A–D), germ cells. Scale bar, 10 µm (A–D); 20 µm (E–L).

In order to address the possible involvement of *sox9b* in sex determination, the expression of sex markers were examined in early stages of gonadal development. *GSDF* [25] and *DMRT1* [20] are typical of male supporting cell markers. In our current experiments, both markers were found to be expressed in all of the homozygous XY gonads examined, but not in the XX gonads (Fig. 2A–H). The female marker, *aromatase*, is known to be expressed only in female somatic gonadal cells including female-specific theca cells [26]. In contrast to *GSDF* and *DMRT1*, *aromatase* was detectable only in XX gonads (Fig. 2I–L). Hence, the genes involved in early sex differentiation in medaka are not regulated by *sox9b*, and sex differentiation therefore proceeds normally at these stages in the *sox9b* mutants.

In the adult heterozygous mutants, the size of ovaries and testes is decreased due to the reduced numbers of germ cells (Fig. S3A–C). We often found the gonads without germ cells (Fig. S3D), revealing a loss of germline maintenance. Interestingly, heterozygous XX mutants often exhibited male secondary sex characteristics (Fig. S3B and S3C) with gonads morphologically comparable to wild-type testes. In these sex-reversed mutants, a testis-marker gene, *P45011β*, is upregulated in the gonad while the expressions of ovarian marker genes, *foxl2* and *aromatase*, were not detected by PCR. Other gonadal markers, which express in both testis and ovary, were detected normally as those in the wild-type gonad (Fig. S3E). The sex reversal phenotype was completely rescued by one copy of *sox9b* transgene in the heterozygous mutant background (Table 1). The heterozygous mutants with female secondary sex characteristics possessed the gonads with ovarian structures having the reduced number of germ cells (Fig. S3B and S3C). We did not observe the sex reversal event that was anticipated given the homology between mammalian *Sox9* and medaka *sox9b*. We also observed no feminization of heterozygous XY mutants (a half-dose of functional *sox9b*) and no masculinization of transgenic XX medaka with an additional *sox9b*-transgene (Table 1), clearly supporting that *sox9b* is not involved in masculinization.

**Table 1:** *Sox9b* mutations lead to a discordance between the genetic and phenotypic sex in the medaka.

*K16X* allele	+/+		+/−		+/−/*sox9b* tg^2^		+/+/*sox9b* tg	
	male^1^	female^1^	male	female	male	female	male	female
**XX**	0	15	7	5	0	14	0	10
**XY**	11	0	7	0	8	0	18	0

*K136X* allele	+/+		+/−		+/−/*sox9b* tg	
	male	female	male	female	male	female
**XX**	0	11	3	5	0	4
**XY**	15	0	15	0	6	0

1 The terms ‘male’ or ‘female’ are defined as secondary sex characteristics and adult gonad morphology (testis or ovary). See also Figure S3.

2 Sox9b tg; one additional copy of *sox9b* transgene.

Very interestingly the compound mutant mice with *Sox8* and *Sox9* revealed a phenotype of gradual loss of germ cells [27]. Therefore with suspect of the conserved *sox9* function on germ cell regulation, we focused on the germ cells in the medaka *sox9b* mutants.

### Reduced proliferation and survival of germ cells in *sox9b* mutants

**Figure 3:**
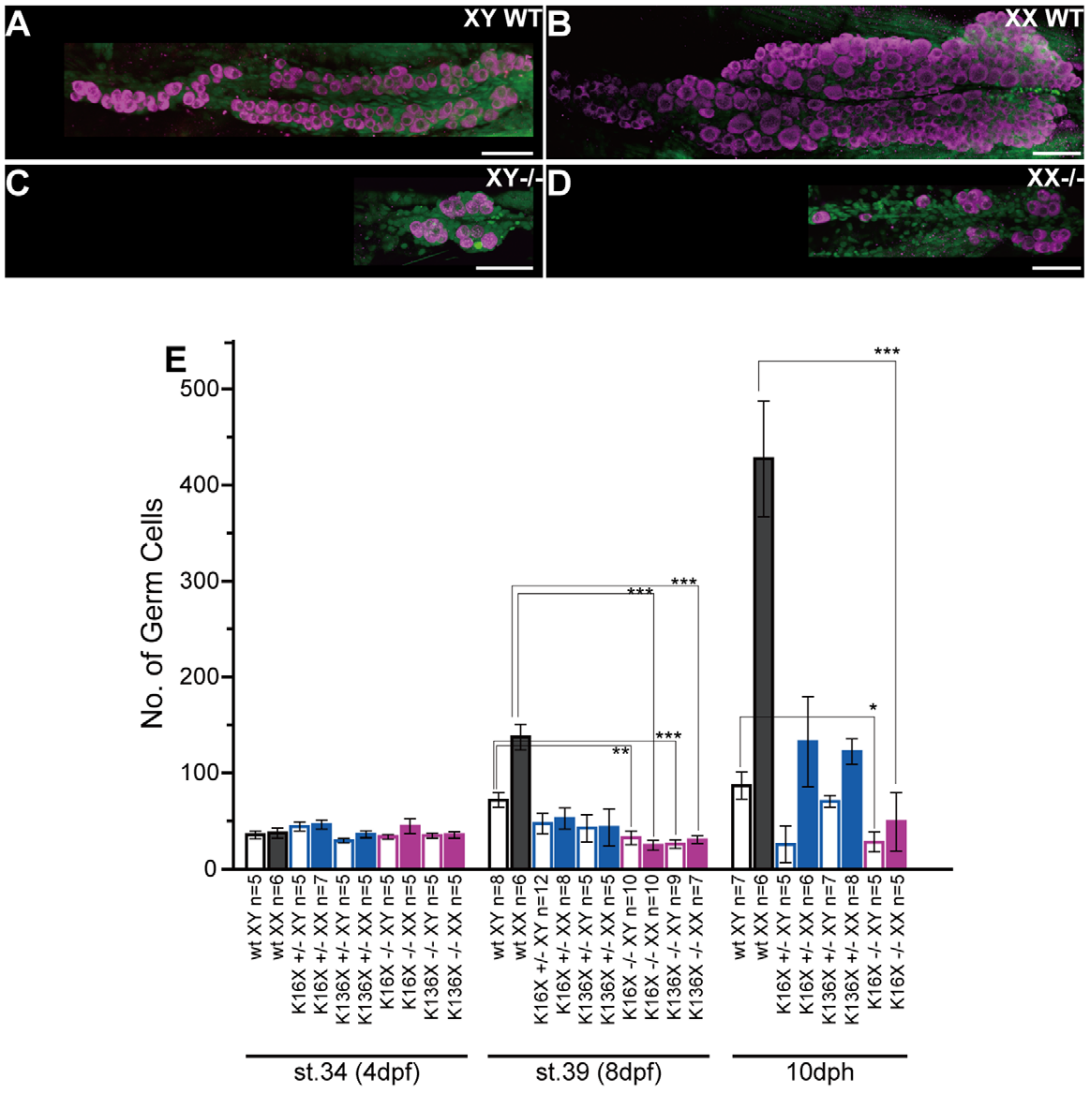
Gonadal morphology and germ cell number in the *sox9b* mutant medaka. (A–D) Ventral images of the medaka gonad at 10 dph obtained by confocal microscopy. Germ cells and nuclei were stained with an anti-OLVAS antibody (purple) and DAPI (green), respectively. The tissue structures are formed but the germ cell numbers are reduced in the mutant gonads. (E) Number of germ cells in wild-type and *sox9b* heterozygous and homozygous mutant medaka during gonad differentiation. *P<0.05, ** *P*<0.01, *** *P*<0.001, Student's *t* test. All values are the mean ± SEM. Scale bars, 50 µm.

In wild-type medaka, the gonads exhibit proliferation of the germ cells with more of these cells found in females as sex differentiation proceeds [28], [29]. At stage 34 (4 dpf) when the gonadal primordium is established with germ cells, no differences were found in the total numbers of germ cells in both wild-type and *sox9b^K16X^* and *sox9b^K136X^* mutant medaka (Fig. 3E and Fig. S4). However, the germ cells in the *sox9b^K16X^* and *sox9b^K136X^* mutant gonads did not increase much at the later stages in either sex. This difference is detectable as early as stage 39 (8 dpf) and becomes more apparent at 10 dph (Fig. 3). The total numbers of germ cells were significantly reduced in these mutants when compared with the wild-type medaka (Fig. 3E). The sexual differences in the germ cell number typically seen in wild-type gonads were also observed in the heterozygous mutants (Fig. 3E and Fig. S4). This indicates that germ cell proliferation and/or survival is affected in *sox9b* mutants.

**Figure 4:**
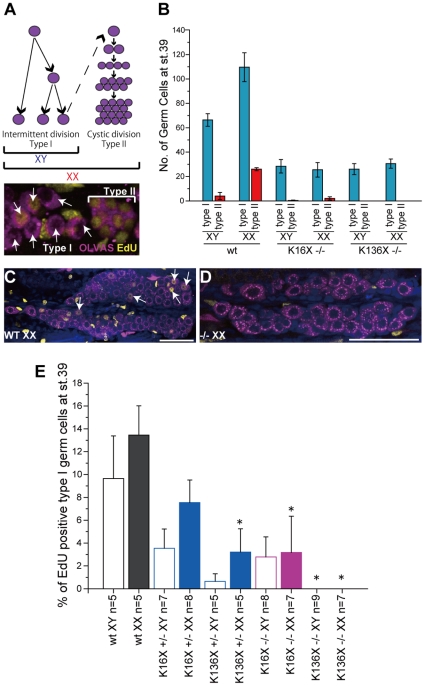
Germ cell proliferation is impaired in the *sox9b* mutant medaka. (A) Medaka exhibit two modes of germ cell division during the early stages of gonadal differentiation. Intermittent divisions (type I) lead to germ cell maintenance and occur in both males and females, whilst synchronous and successive divisions (type II) form germ cell-cysts that are committed to gametogenesis. Type II divisions occur in developing female gonads and cause a female-specific rapid increase in germ cell number. Germ cells undergoing type I or II divisions are identifiable by the presence of isolated (arrows) or packed germ cells (brackets), respectively. (B) In the *sox9b* mutants, germ cells undergoing both type I and II divisions were reduced in number. Cysts containing more than two germ cells were counted as undergoing type II divisions. (C and D) Representative images of EdU labeling experiments in wild-type and mutant medaka gonads at stage 39 are shown. Note that the nuclei of type I germ cells are positively labeled by EdU (yellow) in wild-type (arrows) but not in mutant medaka. Germ cells were stained with an anti-OLVAS antibody (purple). (E) The percentage of EdU-positive type I germ cells was calculated, and type I divisions responsible for germ cell maintenance found to be significantly impaired in the mutants. All values are the mean ± SEM. *P<0.05 student's *t* test (each value was compared with wild-type XY and XX, respectively). Scale bar, 50 µm.

We next characterized the germ cell proliferation modes in the *sox9b* medaka mutants. During the early stages of sex differentiation in the wild-type medaka, there are two modes of germ cell proliferation in operation. The type I mode ensures germ cell maintenance (including stem cell proliferation) which is histologically indicated by the presence of isolated germ cells. Type II proliferation occurs in germ cells that are committed to gametogenesis and involves successive cell divisions. This mode of proliferation results in tightly packed cyst-forming germ cells (Fig. 4A). The female-specific increase in germ cells after the initiation of *DMY*/*dmrt1bY* expression is the consequence of a sub-population of germ cells shifting from type I to type II proliferation [29].

**Figure 5:**
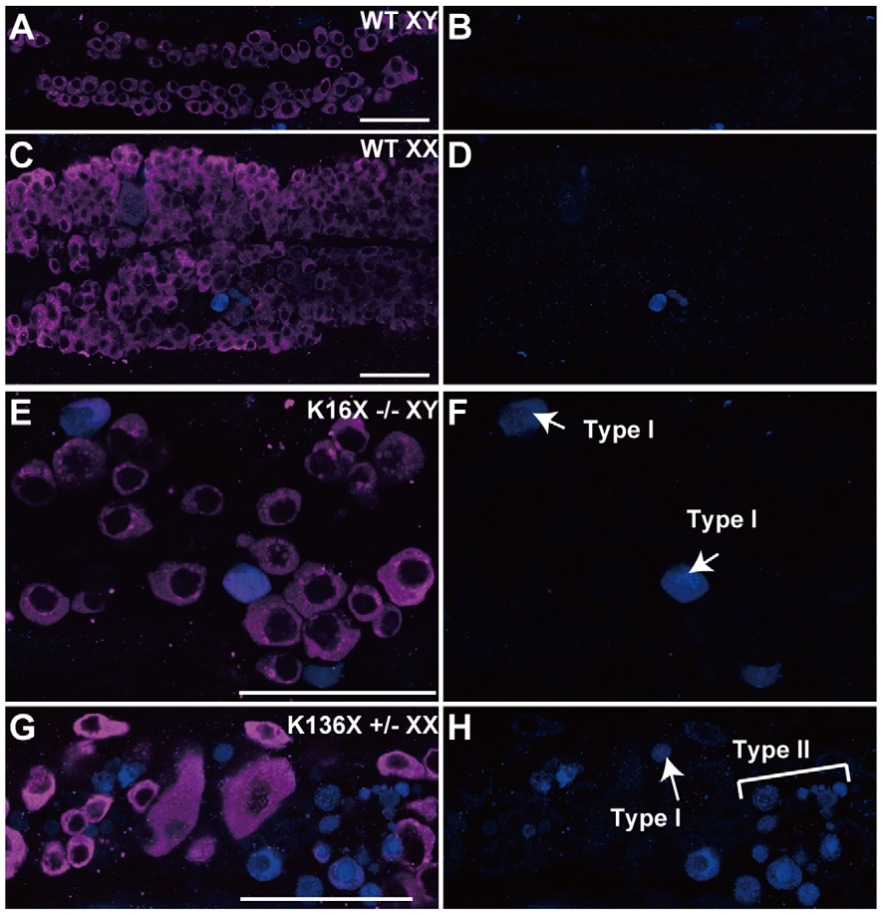
Both type I and II germ cells are eliminated by apoptosis in the *sox9b* mutant medaka. (A–H) Ventral views of 10 dph medaka gonads immunostained with anti-OLVAS (germ cell marker, purple) and anti-cleaved caspase 3 (blue). Merged images (A, C, E and G) and cleaved caspase 3 signals (B, D, F and H) are shown. Low levels of germ cell apoptosis only were evident in wild-type XY and XX medaka at 10 dph. However, type I and type II germ cells (type I, arrows; type II, a bracket) were eliminated by apoptosis (Table 2) in the homozygous (E and F) and heterozygous (G and H) mutants. Scale bar, 50 µm.

The numbers of isolated and cystic germ cells were found not to be increased in the *sox9b^K16X^* and *sox9b^K136X^* medaka mutants during sex differentiation (Fig. 4B). Importantly, we observed that the commitment of germ cells to gametogenesis was not impaired in these mutants as clusters of germ cells undergoing type II division, although quite rare though, were detectable (Fig. 4B). The reduced proliferative activity of type I germ cells was confirmed in the mutants using an EdU incorporation experiment (Fig. 4C–E). Moreover, type I and type II germ cells in the mutant gonads showed frequent apoptosis, as detected by cleaved-caspase3 expression (Fig. 5 and Table 2). Hence, the germ cells in *sox9b^K16X^* and *sox9b^K136X^* mutants demonstrate reduced survival and proliferation. A similar tendency but less severe phenotype was observed in the heterozygous mutants (Fig. 3E, Fig. 4E and Fig. S4).

**Table 2:** Increased apoptotic activity in the germ cells of *sox9b* mutant medaka.

	No. of gonads harboring cleaved-Casp3^+^ germ cells/No. of gonads examined at 10 dph
**wt XY**	0/7 (0%)
**wt XX**	1/6 (17%)
***sox9b^K16X^*** ** +/− XY**	0/5 (0%)
***sox9b^K16X^*** ** +/− XX**	2/6 (33%)
***sox9b^K16X^*** ** −/− XY**	3/5 (60%)
***sox9b^K16X^*** ** −/− XX**	2/5 (40%)
***sox9b^K136X^*** ** +/− XY**	0/7 (0%)
***sox9b^K136X^*** ** +/− XX**	4/8 (50%)

### Cellular associations are impaired in *sox9b* mutants

**Figure 6:**
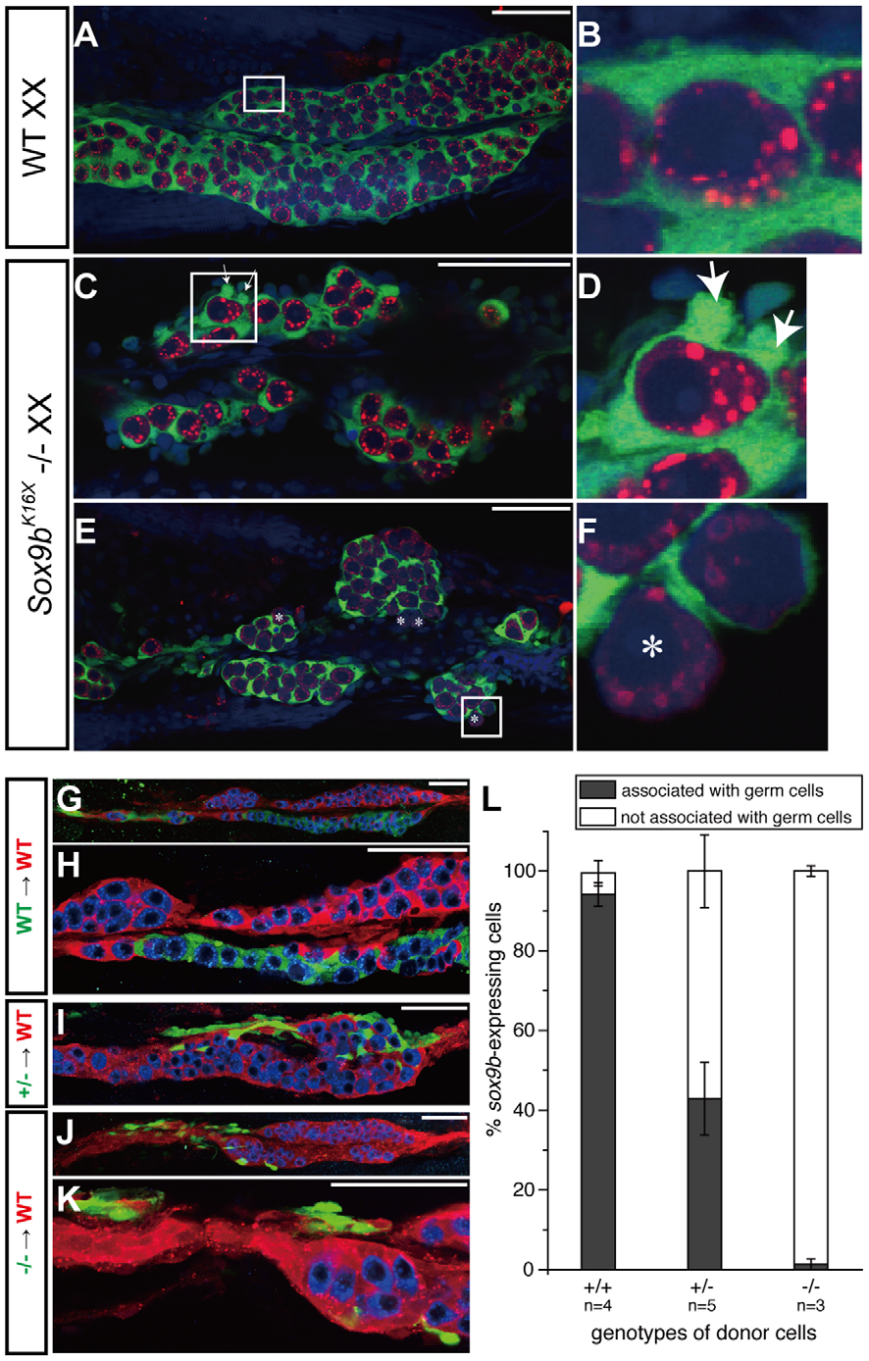
Mutant *sox9b*-expressing cells demonstrate a reduced cellular association. (A–F) The morphologies of wild-type (A and B) and mutant (C–F) medaka gonads. Ventral views of XX gonads at 8 dpf (A–D) and 10 dph (E and F) are shown. Green, *sox9b*-expressing cells were immunostained with anti-GFP; red, germ cells were stained with anti-OLVAS; blue, nuclei were counterstained with DAPI. Medaka germ cells are completely surrounded by *sox9b*-expressing supporting cells and demonstrate a smooth surface (B), whilst mutant *sox9b*-expressing cells have cytoplasmic protrusions (D, arrows). Some isolated germ cells were not completely surrounded by *sox9b*-expressing cells in the mutants (F, asterisks). This was not seen in wild-type animals. (G–K) Representative images of somatic chimera. Green, donor-derived *sox9b*-EGFP expressing cells; red, host *sox9b*-DsRed positive cells; blue, germ cells stained with anti-OLVAS. (L) Calculated ratio of donor-derived *sox9b*-EGFP expressing cells associated with germ cells (black) to those not associated with germ cells (white). Scale bar, 50 µm.

We often observed that the *sox9b*-expressing supporting cells in the *sox9b^K16X^* and *sox9b^K136X^* mutants exhibited aberrant shapes with frequent blebs and incomplete ensheathment (Fig. 6A–F), suggesting an impairment of cell to cell associations leading to abnormal germ cell homeostasis. To further evaluate these effects, chimeric gonads containing both wild-type and mutant cells were generated via transplantation. In contrast to chimeric gonads between wild-type cells, mutant *sox9b*-expressing supporting cells tended to be expelled from the chimeric gonads (Fig. 6G–K). The frequency of contact between *sox9b*-expressing supporting cells and germ cells was found to be dramatically decreased (Fig. 6L). In addition, homozygous mutant cells exhibited a more severe phenotype than heterozygous mutant cells, indicating that this severity depends on the functional *sox9b* gene dose per supporting cell. Since mutant *sox9b*-expressing supporting cells retain the ability to form gonads, the phenotype of the chimeric gonads can be attributed to a reduced capability of the mutant *sox9b*-expressing supporting cells to associate with each other, but not to a loss of identity of the supporting cells.

**Figure 7:**
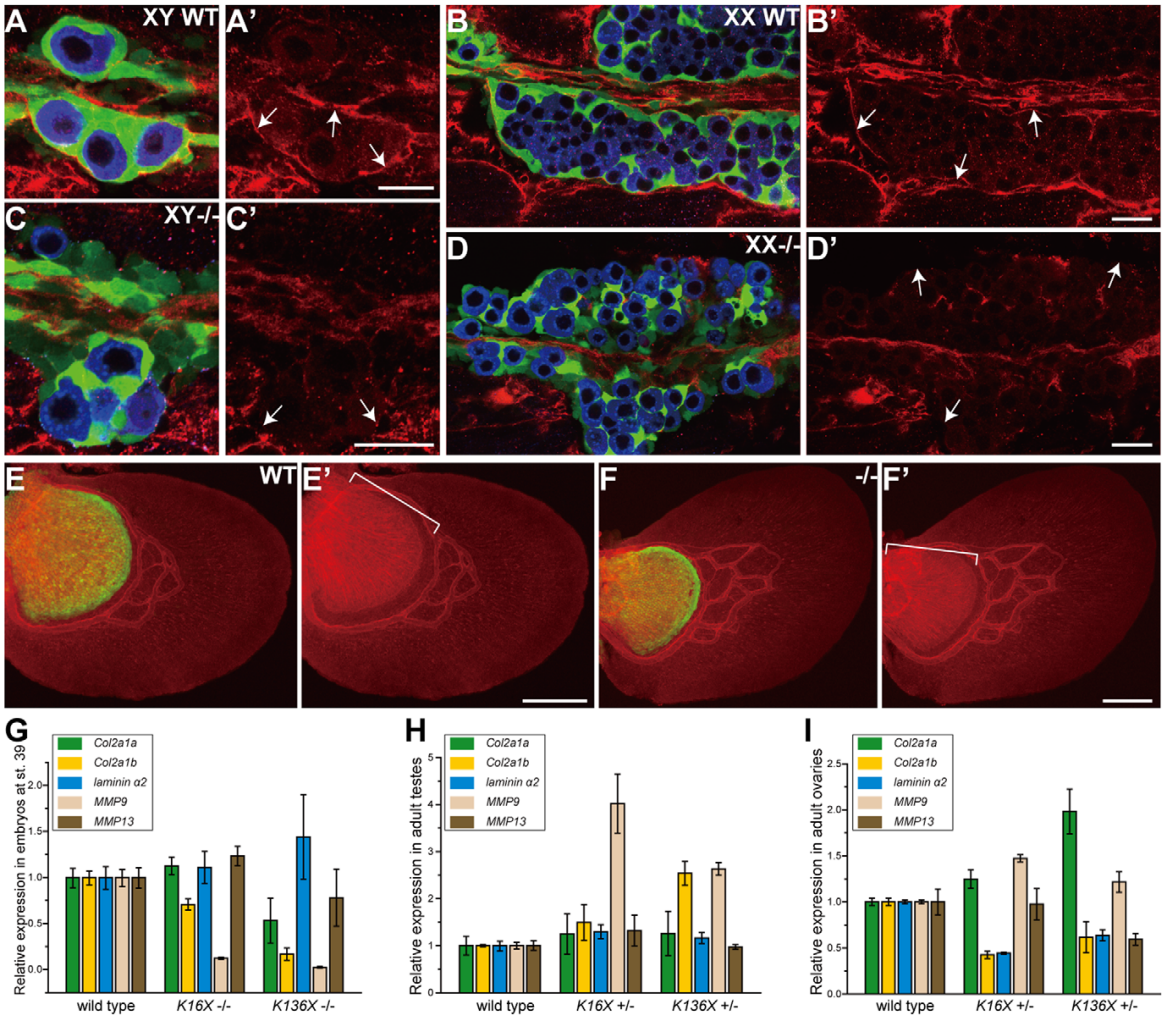
The deposition of ECM components is altered in *sox9b* medaka mutants. (A–D′) Ventral views of wild-type and *sox9b^K16X^*−/− medaka gonads at stage 39. Laminin (red), *sox9b*-EGFP (green) and germ cells detected by OLVAS antibody (blue) are shown. Note that the laminin deposits detected in wild-type *sox9b*-expressing cells are disorganized in the mutant cells (A′, B′, C′ and D′, arrow). (E–F′) The wild-type and *sox9b^K16X^*−/− pectoral fin at stage 39. Laminin (red) and *sox9b*-EGFP (green) signals are shown. The laminin expression pattern (brackets in E′ and F′) is unchanged in the mutant pectoral fin, in which both *sox9a* and *sox9b* are expressed. (G–I) The relative mRNA expression levels of extracellular matrix (ECM) components and matrix metalloproteases (MMP) in stage 39 medaka embryos (G), and the adult testis (H) and ovary (I) determined by qRT-PCR. The intensity of each band was normalized to EF1α and the relative expression levels compared with wild type are shown. Note that some ECM components and MMP are upregulated or downregulated in mutants compared with wild type. Each value represents the mean ± s.e.m. (n = 4). Scale bars, 20 µm and 100 µm (E–F′).

A number of studies have attributed many aspects of *Sox9*-involving phenomena to the regulation of extracellular matrices [1], [4], [30]. In medaka, we observed that laminin deposition was abnormal in the *sox9b* mutant gonad (Fig. 7A–D) whereas the tissues expressing both *sox9a* and *sox9b* do not show this defect (Fig. 7E, 7F). Furthermore, the expression of other extracellular matrix components, such as *collagen* genes and *MMP* genes, were found to be altered in mutant embryos, testes and ovaries (Fig. 7G–I). Hence, the observed impairment of cellular associations via the disorganization of extracellular matrix components is consistent with the failure of germ cell maintenance in medaka *sox9b* mutants, although further analysis needs to be done to fully elucidate these pathways.

### The increasing number of germ cells rescues the masculinization phenotype in *sox9b* mutants

The seemingly paradoxical masculinization of the medaka *sox9b* mutants is consistent with the germ cell-loss phenotype found in adult mutant gonads because our previous analysis has indicated that gonadal somatic cells, in the absence of germ cells, are predisposed to male development (Fig. S3F) [31], [32]. In contrast, the *hotei* homozygous mutant has a defect in the gene expressing the type II receptor for the anti-Müllerian hormone (*amhrII*) and shows continuous proliferation of germ cells [33]. Since the receptor and the ligand (anti-Müllerian hormone) are both expressed in the supporting cells but not in the germ cells, the impairment of anti-Müllerian hormone system consequently causes the germ cell-excessive phenotype. The homozygous *hotei* mutants show the male to female sex reversal phenotype but the sex reversal is not observed in the heterozygous *hotei* mutants. In this context, the number of germ cells in the gonad affects the proper sex differentiation directed by the sex chromosomes, and wild-type XX medaka without germ cells develop male secondary sex characteristics with a male gene expression profile (Fig. S3F).

To further obtain the solid evidence that the masculinization in *sox9b* mutants is a secondary effect of the decreasing number of germ cells, we crossed a *sox9b* mutant with a *hotei* heterozygous mutant to recover the germ cell number. We found that the number of germ cells is recovered and that the female to male sex reversal phenotype of the *sox9b* heterozygous XX mutants was abolished completely in the *hotei* and *sox9b* compound heterozygous mutants (Table 3). This result indicates that the female to male sex reversal phenotype of the *sox9b* mutants is caused by a decreased number of germ cells and not by a direct impairment of *sox9b*-expressing supporting cells. Taken together with the gene expression analysis and the analysis of medaka containing different doses of functional *sox9b* gene, we conclude that medaka *sox9b* is not required for testis determination or the subsequent processes of early testis differentiation.

**Table 3:** Female to male sex reversal phenotype is rescued in *sox9b* and *hotei* compound heterozygous mutants.

	*amhrII^hot^* +/−		*sox9b^K16X^*+/−;*amhrII^hot^* +/−	
	male	female	male	female
**XX**	0	4	0	14
**XY**	9	0	9	0

## Discussion

We find in our current study that the medaka *sox9b* gene does not directly regulate testis determination and differentiation in this teleost species but is involved in germline maintenance and survival in both female and male gonads. The combined expression domains of medaka and zebrafish *sox9a* and *sox9b* largely match those of mouse *Sox9*, suggesting that the conserved *sox9* roles among vertebrates are divided between two *sox9* genes in medaka as a result of teleost-specific genome duplication. Medaka heterozygous *sox9b* mutants are viable and a few homozygous mutants can survive until 20 dph. This is in contrast to the embryonic lethality seen in the *Sox9*-disrupted mouse embryo. The discrepancy in these survival outcomes between medaka and mouse could be attributable to the redundant expression of *sox9a* and *sox9b* in the medaka.

### A testis-determining role of mammalian *Sox9* is likely to be neofunctionalized

During chondrogenesis, mammalian *Sox9* directly regulates the expression of *Col2a1*, the extracellular matrix component type II collagen gene [34]. In mouse chimeras, *Sox9* ^−/−^ mesenchyme cells are excluded from wild-type cells during cartilage formation, possibly due to a disorganized extracellular matrix [35]. It is intriguing that a gradual loss of germ cells has been reported in the compound *Sox8* and *Sox9* mutant mouse and concluded to be the result of functionally defective supporting cells caused by the loss of *Sox8* and *9* redundant activity [27]. This is a representative example of the germ cell maintenance role of *Sox9* in mammalian gonads. Our present results show that the germ cell-loss in our medaka *sox9b* mutants are likely to have been caused by reduced cellular associations. The results of these studies are therefore collectively supportive of an evolutionally conserved role of *sox9* in the regulation of germ cell maintenance and survival. In this context, both medaka *sox9a* and *sox9b* seem to have a redundant role on the regulation of extracellular matrix because the laminin deposition in the pectoral fin was not impaired in the *sox9b* mutants (Fig. 7E, 7F).

Interestingly, medaka *sox9b* mutants show a female to male sex reversal phenotype, which is the opposite effect of the masculinizing function of *Sox9* in the mammalian gonads. Our current results demonstrate that the masculinization caused by the loss of *sox9b* function is accounted for by a decreased number of germ cells. Recent comparative genomic approaches have revealed that testis-specific enhancer of *Sox9* core element (TESCO), which is a binding region for *Sry* and is critical for the male-specific expression of *Sox9* in supporting cells in the mouse, is conserved in amniotes and amphibia (*Xenopus tropicalis*), but not in the medaka or zebrafish [36]. This provides a fascinating view that TESCO-mediated regulation was utilized by the evolutional stem from amphibia and was then recruited to mediate the testis-determining function of *Sox9* in parallel with the emergence of *Sry* in mammals. This possibility is consistent with our current data showing that a sex determining gene-*sox9* axis does not underpin sex determination in medaka and suggests that *Sox9* is likely to play a neofunctionalized role in testis determination in mammals, which is appended to the more conserved *sox9* role in germ cell maintenance.

It is also of interest that the loss of *Sox9* expression in mammalian female supporting cells may be related to the lack of premeiotic germ cells in the adult ovary. This would explain why germline stem cells are either absent or very few in number in the mammalian ovary and also the species-specific configuration of gonadal differentiation.

In conclusion, the molecular pathways in medaka that start with *DMY*/*dmrt1bY* represent a unique system of testis determination and differentiation and should prompt a reconsideration of the discussion regarding the *sox9*-dependent testis determination system in many vertebrates.

## Materials and Methods

### Ethics Statement

All the treatments of animals in this research followed the guideline of National Institute for Basic Biology and were approved by the Institutional Animal Care and Use Committee of National Institutes of Natural Sciences. The approval IDs by the committee are 11A094 and 10A023.

### Medaka strains and isolation of tilling medaka

The wild-type cab strain, *Sox9b*-EGFP/DsRed transgenic medaka [20], [37] and *hotei* mutants [33] were used in this study. Isolation of tilling medaka was performed as previously described by Taniguchi et al. [24]. The region encompassing the first and second exons of the medaka *sox9b* gene, including the initial ATG, was screened for mutations using the primers 5′-AACTCTTGGACGCAGAAAGG-3′ (forward) and 5′-TCAGGGTGCAAACGGATAAC-3′ (reverse). The PCR products were treated with ExoSAP-IT (GE Healthcare) and sequenced using the forward primer and a 3730xl 96-capillary DNA analyzer (Applied Biosystems). Two different alleles (*K16X* and *K136X*) were identified, as shown in Figure 1A. Medaka that are homozygous for the *K16X* or *K136X* alleles die by 20 dph (days post hatching).

### Genotyping

The sex of the fish (XY or XX) was determined by PCR genotyping using a previously described method to detect polymorphisms between *dmy* and *DMRT1* [20]. To genotype the *sox9b* alleles (+/−, +/− or −/−), direct sequencing and/or SNP genotyping assays were performed. For direct sequencing, genomic regions encompassing the mutation sites were amplified by PCR using the primers; *sox9b tilling F* 5′-GGGCTCCAACTCTTGGACGC-3′ and *sox9b tilling R* 5′-CAATAAAACCTCGTGCGCCG-3′. After removing dNTPs by ExoSAP-IT (USB), amplified fragments were used as templates and subjected to sequencing by PCR with the forward or reverse primers described above. Sample sequences are shown in Figure 1B. Genotyping of *sox9b* mutant alleles was also performed using a custom SNP genotyping assay (Applied Biosystems) and a real time PCR system (StepOne; Applied Biosystems). The following primers and TaqMan probes were used for the SNP genotyping assay:

*Sox9b^K16X^*

*seq_sox9b_F* 5′-CCTCGATCCATACCTGAAGATGACA-3′

*seq_sox9b_R* 5′-ACTGGGAGCGTCGGAGT-3′

*seq_sox9b_VIC* 5′-VIC-AAGAACAGGAGAAGTGTC-3′ (wild type)

*seq_sox9b_FAM* 5′-FAM-AAGAACAGGAGTAGTGTC-3′ (mutant)

*Sox9b^K136X^*

s*eq_sox9b_F* 5′-CCAATACCCGCATTTGCACAAC-3′

*seq_sox9b_R* 5′-GGGCTTACCTCCAAAGTTTTCCA-3′

*seq_sox9b_VIC* 5′-VIC-AGCTCAGCAAAACT-3′ (wild type)

*seq_sox9b_FAM* 5′-FAM-CAGAGCTCAGCTAAACT-3′ (mutant)

### Western blotting

Western blotting was performed as described previously [38]. Briefly, after SNP genotyping using the fins of *sox9b^K16X^* and *sox9b^K16X^* offspring at stage 39, each embryo (+/+, +/− and −/−) was crushed and boiled in SDS sample buffer (62.5 mM Tris-HCl pH 6.8, 2% SDS). Following centrifugation, the supernatant was used for western blotting. Anti-Sox9b serum was raised in rabbits immunized with a sox9b peptide (RAQYDYSDHQNSANS). Anti-sox9b serum (rabbit, 1/500) and an alpha tubulin antibody (mouse, 1/4000; Sigma) were used as the primary antibodies. The secondary antibodies used included anti-rabbit HRP (1/2000; Zymed) and anti-mouse HRP (1/2000; Nacalai).

### *In situ* hybridization, immunohistochemistry and histology

*In situ* hybridization (ISH), immunohistochemistry (IHC) and histology were performed as previously described [39]. *Sox9b^K16X^* and *sox9b^K16X^* siblings were collected and fixed with 4% PFA at stage 34, stage 39 and 10 dph. For ISH, a medaka *GSDF* clone (NCBI accession number; FS532259) was obtained from the NBRP Medaka cDNA library (http://www.shigen.nig.ac.jp/medaka/top/top.jsp) and used as the RNA probe. RNA probes for *DMRT1* and *aromatase* were prepared as described previously [20], [26]. Zebrafish *sox9a* and *sox9b* probes were gifts from Dr. C. Chung and were prepared as described [22]. Genetic sex and *sox9b* genotypes were determined after ISH. Transverse plastic sections (4 µm) were also prepared as described [39]. For IHC, anti-GFP (1/100, rat; Nacalai or 1/100, mouse; Clontech), anti-DsRed (1/100, rabbit; Clontech), anti-OLVAS (1/100, rat) [38], anti-cleaved caspase3 (1/100, rabbit; Cell Signaling Technology) and anti-laminin (1/100, rabbit; SIGMA) were used as the primary antibodies followed by incubation with Alexa 488, 568 or 647 coupled secondary antibodies (1/100, goat; Molecular Probes).

### EdU incorporation experiments

Germ cell proliferation was assessed using the Click-iT EdU labeling kit (Invitrogen). Embryos of *sox9b^K16X^* and *sox9b^K16X^* offspring at stage 39 were exposed to 500 µM EdU/BSS (Balanced Salt Solution) for 1 h and then fixed in 4% PFA. Detection of EdU was performed according to the manufacturer's instructions. After genotyping, IHC using anti-OLVAS (rat, 1/100) and anti-rat Alexa 488 (1/100; Molecular Probes) was performed. Embryos were counterstained with DAPI and EdU positive germ cells/all germ cells ratios were determined in both the wild type and mutants.

### Generation of chimeric medaka

Medaka embryos from wild-type *sox9b*-DsRed and the *sox9b^K16X^* (+/−)/*sox9b*-EGFP incross were used as the host and donor, respectively. To mark the donor cells, 1% fluorescent dextran (Fluroruby; Molecular Probes) was microinjected into donor embryos at the one cell stage. Donor and host embryos at the mid-blastula stage were dechorionated in a solution containing hatching enzyme (obtained from NBRP medaka) for 30 min. Pipettes for transplantation were prepared by pulling 1 mm glass capillary tubes using a PC-10 puller (Narishige). Using a pipette installed on an oil-driven manipulator (CellTram Vario; Eppendorf), the labeled donor cells were transferred into the dechorionated mid-blastula stage host embryos on an 0.8% agar plate filled with 0.9% BSS (balanced salt solution). One day after transplantation, donor embryos were genotyped to distinguish +/+, +/− or −/−. Chimeric embryos were incubated up to stage 39 and fixed in 4% PFA. IHC was performed using anti-GFP (rat, 1/100; Nacalai), anti-DsRed (rabbit, 1/100; Clontech) and anti-OLVAS (rat, 1/100) antibodies.

### *Sox9b* rescue experiments

The *sox9b* coding sequence lacking its stop codon (*Sox9b* CDS) was inserted into the *Sal*I site of the pBLSK+ PTV1-2A-mCherry vector [40], (a gift of Dr. Hibi; Nagoya University), upstream of the 2A peptide. The resulting *sox9b* CDS- PTV1-2A -mCherry DNA fragment was then inserted by homologous recombination into a previously described BAC clone containing the entire *sox9b* gene [20], [37]. This modified BAC was then injected into fertilized eggs of the OKcab strain and two independent stable lines were obtained. One line was used for rescue experiments by crossing with *sox9b* mutants. The *Sox9b* transgene copy numbers were determined by qPCR with THUNDERBIRD SYBR qPCR Mix (TOYOBO) using primers for *sox9b* and *dmy* (one copy control). The following primers were used for qPCR;

*q-sox9b-F*, 5′-TTCCTTACGCACGATCCTCA-3′;

*q-sox9b-R*, 5′-TTCGATCTTTCACTGGTTTGC-3′;

*q-dmy-F*, 5′-CTCCGGTAAATTGACGCACA-3′;

*q-dmy-R*, 5′-GTCTGACTTTCCGGTCAAAGG-3′.

### RT-PCR analysis of heterozygous mutant gonads

RT-PCR was performed as previously described [38]. Briefly, total RNA from the ovaries or testes of wild type or *sox9b* heterozygous mutants was isolated by ISOGEN (Nippon Gene). After removing genomic DNA by treatment with DNase I (Ambion), single stranded cDNA was synthesized from 300 ng total RNA using the SuperScript III First-Strand Synthesis System (Invitrogen). The PCR reactions were performed using the primers described above [31].

### qRT-PCR analysis of ECM and MMP genes

Total RNA was prepared from wild-type or *sox9b* mutant embryos, adult testes and ovaries using ISOGEN (Nippon Gene) or RNAqueous (Ambion). After removal of the genomic DNA by treatment with DNase I (Ambion), cDNA was prepared from a 300 ng aliquot of total RNA using SuperScript III First-Strand Synthesis System (Invitrogen, Carlsbad, CA) and used for subsequent qPCR analysis. qPCR. was performed using the Thunderbird SYBR qPCR mix (Toyobo) and StepOne (ABI) and the primers *col2a1a* F, 5′- GGCAACAGCCGCTTTACTTA -3′ and *col2a1a* R, 5′- AATGTCCACAATGGGCAAAC -3′; *col2a1b F*, 5′- GGTAACAGCCGCTTCACCTA -3′ and *col2a1b* R, 5′- AATGTCCATGGGAGCAATGT -3′; *laminin α2 F*, 5′- CCCAATCTACGTGGGAGGAT -3′ and *laminin α2 R*, 5′- GTCTTTGACGCCTTGGTGAT -3′; *MMP13 F*, 5′- AGGTCGATGCTGCTGCTTAC -3′ and *MMP13 R*, 5′- GCATTCAAGGATGGAGTTGG -3′; *MMP9 F*, 5′- TTGACAAAGGCTACCCCAAG -3′ and *MMP9 R*, 5′- CCGCCAGTAGAATTGGTCAC -3′; *EF1α F*, 5′- CATGGTTGTGGAGCCTTTCT -3′ and *EF1α R*, 5′- CTTTCTCTGCAGCCTTGGTC -3′.

## Supporting Information

Figure S1 **Syntenic analysis and expression study of medaka** ***sox9*** **genes.** (A) Syntenic analysis was performed using *Ensembl* genome browsers among mouse, chick, frog (*Xenous tropicalis*), zebrafish, stickleback and medaka. (B) Medaka *sox9a* (right) and *sox9b* (left) expression in XY gonads at stage 39. Asterisks indicate germ cells. Scale bars, 20 µm.
(TIF)

Figure S2 **Zebrafish** ***sox9a*** **and** ***sox9b*** **expressions in adult ovaries and testes.** (A–C) *Sox9a* expression in zebrafish adult ovaries (A and B) and a testis (C). B is a higher magnification view of the inset in A. Note that *sox9a* was expressed in the some parts of somatic cells surrounding small germ cells in the adult ovary. (D and E) *Sox9b* expression in a ovary and a testis. *Sox9b* is detected only in oocytes but not in testis. Signals are indicated as arrows. Scale bars, 50 µm.
(TIF)

Figure S3 **Phenotypes of adult heterozygous mutant medaka.** (A–C) Fin shapes (left) and transverse sections of adult gonads (right) in wild-type (A), *sox9b^K16X^* +/− (B) and *sox9b^K136X^* +/− (C) medaka. Wild-type XY medaka display a jagged dorsal fin and a sharp anal fin, which is typical male secondary sex characteristics. Round-shaped dorsal and anal fins and a developed urinogenital papilla (arrows) are characteristic of wild-type XX medaka. Alleles (*K16X* or *K136X*), genetic sex (XY or XX) and phenotypic sex (male or female) are indicated on the left of each panel (B and C). Some XX heterozygous mutants showed female to male sex reversal for both secondary sex characteristics and gonad morphology (middle panels in B and C). (D) A representative image of a germ cell-deficient gonad in an XX heterozygous medaka mutant. This mutant exhibited male secondary sex characteristics. (E) Expression of several sex-related genes assessed by RT-PCR in wild-type and heterozygous mutant gonads. The gene expression patterns in the gonads of XX male heterozygous mutants are consistent with those of the wild-type XY gonads. (F) The sex of the medaka is determined by the presence or absence of the Y chromosome. However sex differentiation requires proper homeostasis of the germ cells. Germ cell-deficient medaka exhibit female to male sex reversal of secondary sex characteristics independently of the genetic sex. Fewer germ cells are inclined to produce a male phenotype whereas hypertrophic germ cells, as in the *hotei* mutant, cause a male to female sex-reversal phenotype. Female to male sex reversal in heterozygous *sox9b* mutants is explained by the secondary effects of a reduced number of germ cells but not by the direct effects of *sox9b*-expressing cell impairment. Scale bar, 500 µm.
(TIF)

Figure S4 **Ventral images of medaka wild-type and mutant gonads at different stages.** (A–C) Ventral views of medaka gonads at the stage of gonadal primodium, stage 34 (A), the stage of female-specific increase of germ cells, stage 39 (B) and the stage of apparent sexual dimorphism of gonads, 10 dph (C). The germ cells and nuclei were immunostained with OLVAS (purple) and DAPI (green), respectively. Images from wild-type (upper), heterozygous (middle) and homozygous (lower) *sox9b^K16X^* medaka are shown. Scale bar, 20 µm (A) and 50 µm (B and C). n, number of gonads examined.
(TIF)
