# Supplementary figures and images for: Analysis of Medaka sox9 Orthologue Reveals a Conserved Role in Germ Cell Maintenance
Source: PLoS One. 2012 Jan 12;7(1):e29982. doi: 10.1371/journal.pone.0029982 (PMC3257256; doi:10.1371/journal.pone.0029982)

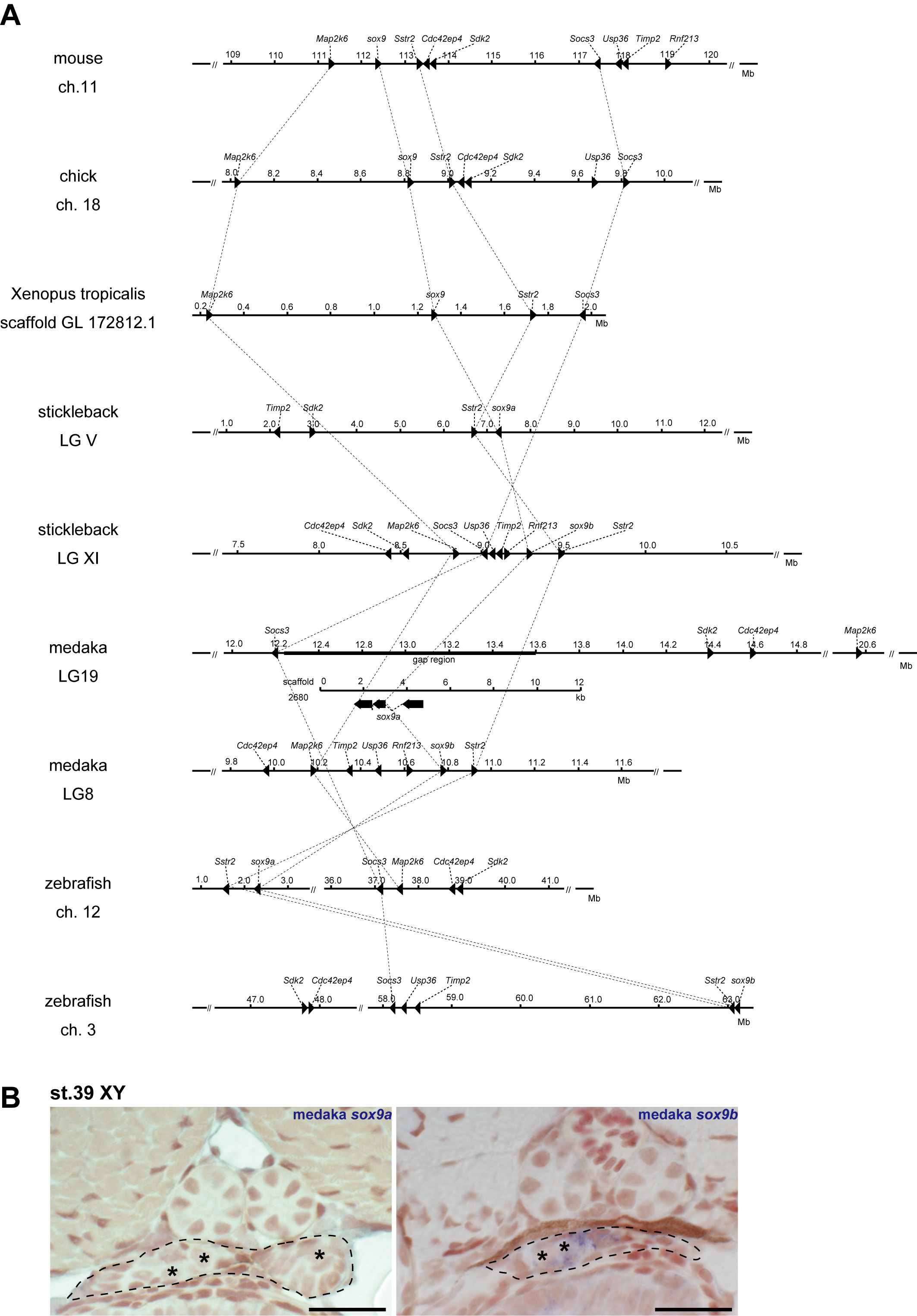

Supplement: Figure S1 — Syntenic analysis and expression study of medaka sox9 genes. (A) Syntenic analysis was performed using Ensembl genome browsers among mouse, chick, frog (Xenous tropicalis), zebrafish, stickleback and medaka. (B) Medaka sox9a (right) and sox9b (left) expression in XY gonads at stage 39. Asterisks indicate germ cells. Scale bars, 20 µm. (TIF) [file pone.0029982.s001.tif]

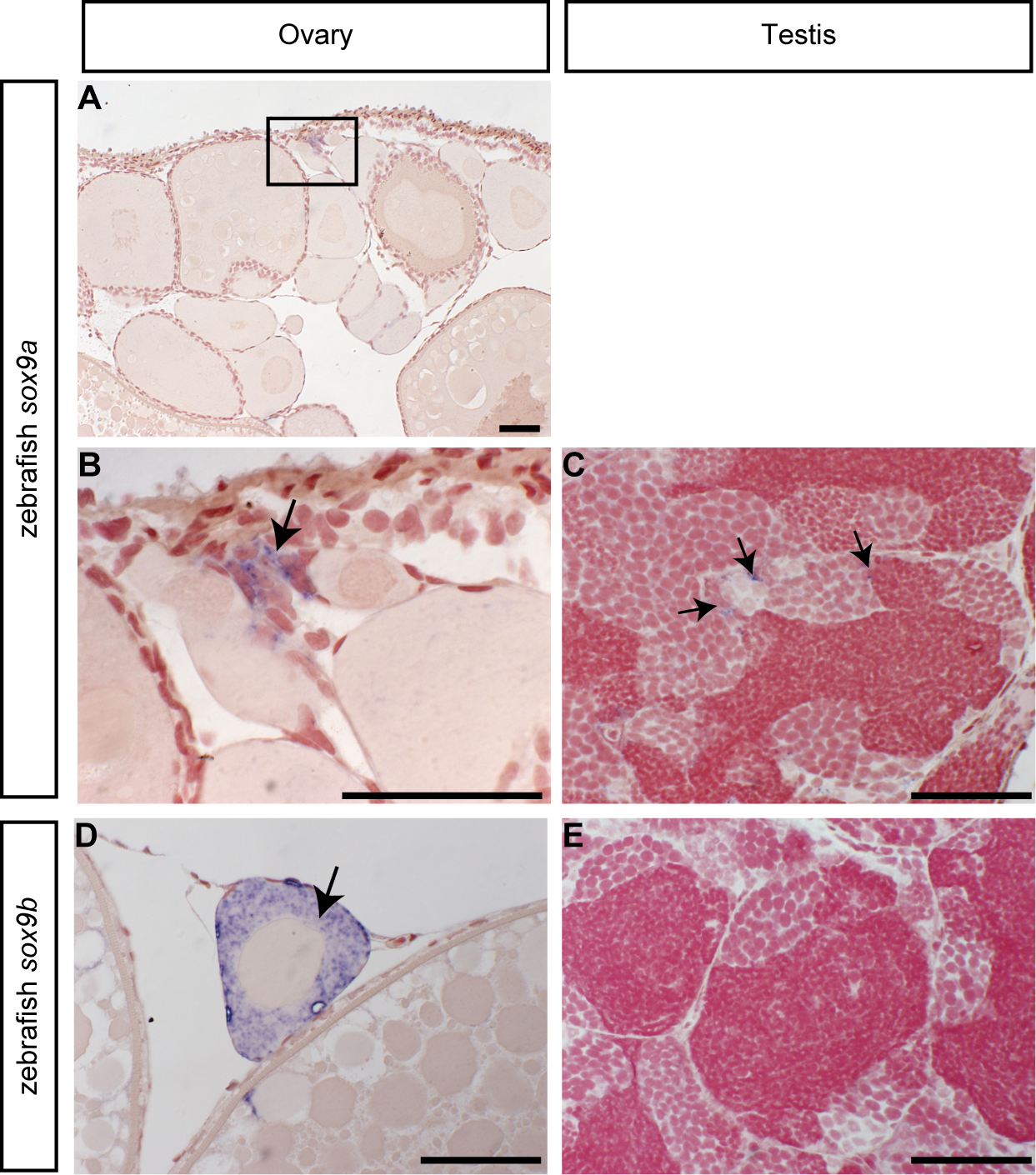

Supplement: Figure S2 — Zebrafish sox9a and sox9b expressions in adult ovaries and testes. (A–C) Sox9a expression in zebrafish adult ovaries (A and B) and a testis (C). B is a higher magnification view of the inset in A. Note that sox9a was expressed in the some parts of somatic cells surrounding small germ cells in the adult ovary. (D and E) Sox9b expression in a ovary and a testis. Sox9b is detected only in oocytes but not in testis. Signals are indicated as arrows. Scale bars, 50 µm. (TIF) [file pone.0029982.s002.tif]

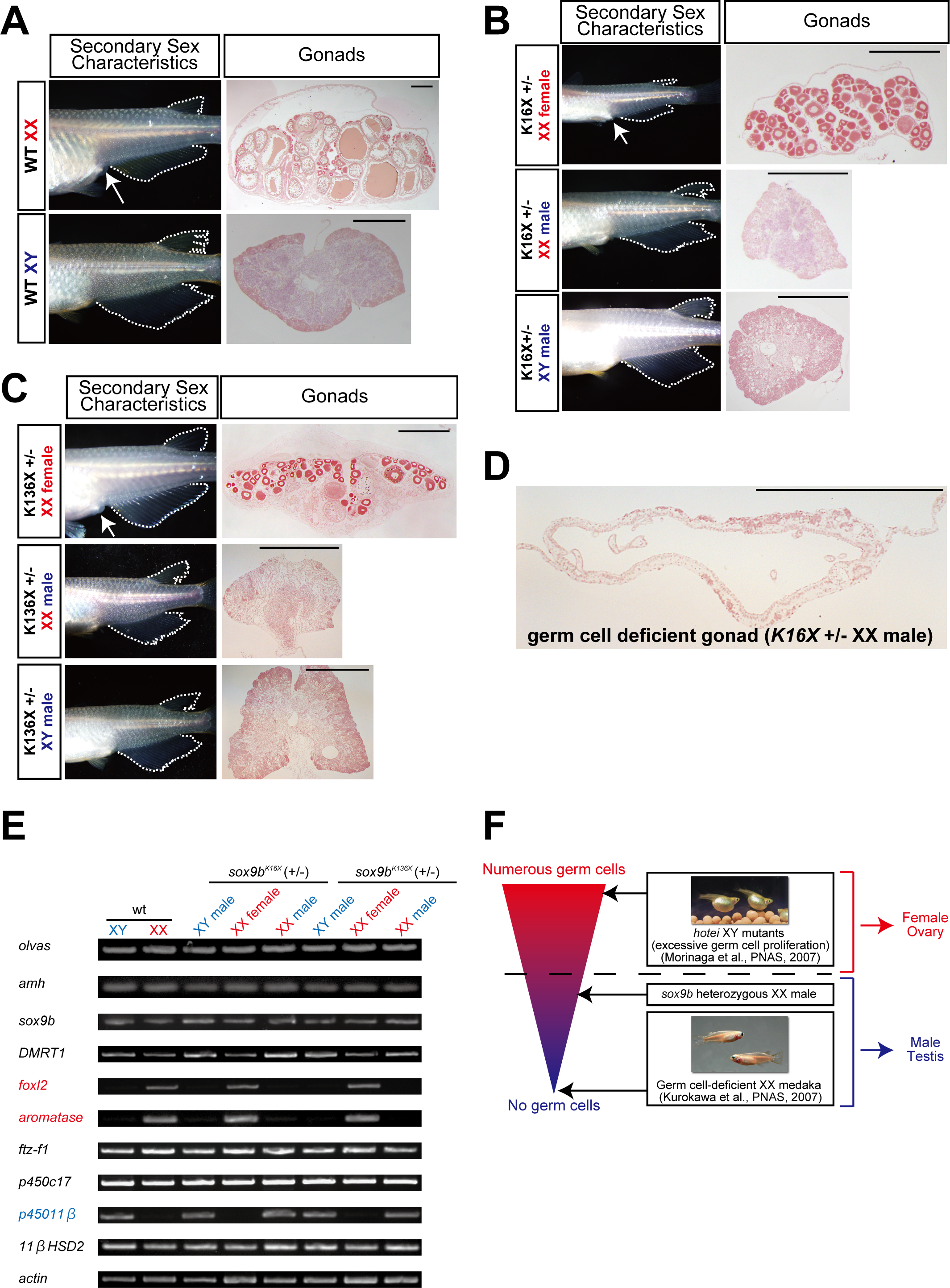

Supplement: Figure S3 — Phenotypes of adult heterozygous mutant medaka. (A–C) Fin shapes (left) and transverse sections of adult gonads (right) in wild-type (A), sox9bK16X +/− (B) and sox9bK136X +/− (C) medaka. Wild-type XY medaka display a jagged dorsal fin and a sharp anal fin, which is typical male secondary sex characteristics. Round-shaped dorsal and anal fins and a developed urinogenital papilla (arrows) are characteristic of wild-type XX medaka. Alleles (K16X or K136X), genetic sex (XY or XX) and phenotypic sex (male or female) are indicated on the left of each panel (B and C). Some XX heterozygous mutants showed female to male sex reversal for both secondary sex characteristics and gonad morphology (middle panels in B and C). (D) A representative image of a germ cell-deficient gonad in an XX heterozygous medaka mutant. This mutant exhibited male secondary sex characteristics. (E) Expression of several sex-related genes assessed by RT-PCR in wild-type and heterozygous mutant gonads. The gene expression patterns in the gonads of XX male heterozygous mutants are consistent with those of the wild-type XY gonads. (F) The sex of the medaka is determined by the presence or absence of the Y chromosome. However sex differentiation requires proper homeostasis of the germ cells. Germ cell-deficient medaka exhibit female to male sex reversal of secondary sex characteristics independently of the genetic sex. Fewer germ cells are inclined to produce a male phenotype whereas hypertrophic germ cells, as in the hotei mutant, cause a male to female sex-reversal phenotype. Female to male sex reversal in heterozygous sox9b mutants is explained by the secondary effects of a reduced number of germ cells but not by the direct effects of sox9b-expressing cell impairment. Scale bar, 500 µm. (TIF) [file pone.0029982.s003.tif]

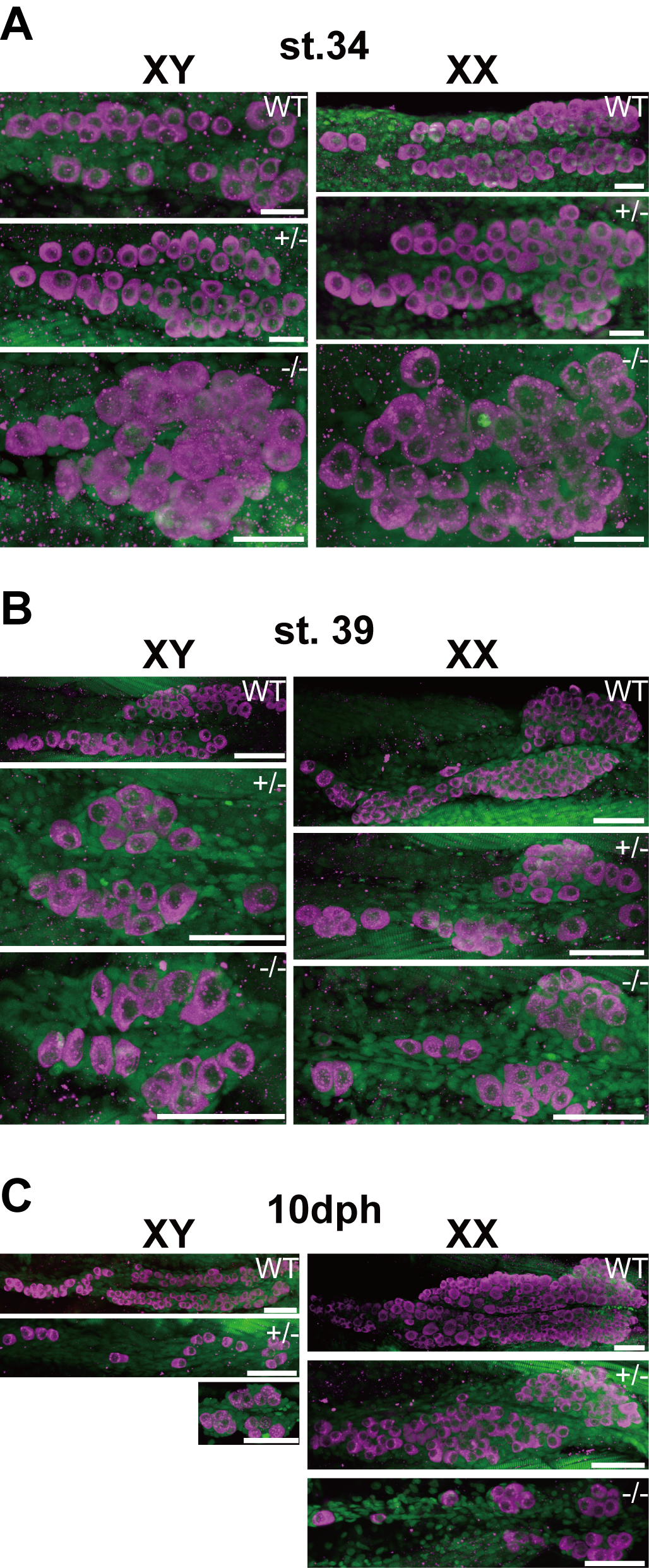

Supplement: Figure S4 — Ventral images of medaka wild-type and mutant gonads at different stages. (A–C) Ventral views of medaka gonads at the stage of gonadal primodium, stage 34 (A), the stage of female-specific increase of germ cells, stage 39 (B) and the stage of apparent sexual dimorphism of gonads, 10 dph (C). The germ cells and nuclei were immunostained with OLVAS (purple) and DAPI (green), respectively. Images from wild-type (upper), heterozygous (middle) and homozygous (lower) sox9bK16X medaka are shown. Scale bar, 20 µm (A) and 50 µm (B and C). n, number of gonads examined. (TIF) [file pone.0029982.s004.tif]
